# “Our Patients Are Different”: Predictors of Seclusion and Restraint in 31 Psychiatric Hospitals

**DOI:** 10.3389/fpsyt.2022.791333

**Published:** 2022-04-26

**Authors:** Erich Flammer, Sophie Hirsch, Nancy Thilo, Tilman Steinert

**Affiliations:** ^1^Clinic for Psychiatry and Psychotherapy I, Ulm University, Ulm, Germany; ^2^Centres for Psychiatry Suedwuerttemberg, Ravensburg, Germany

**Keywords:** seclusion, restraint, coercion, structural characteristics, hospital characteristics, patient characteristics

## Abstract

**Background:**

Research in recent years has demonstrated that the use of coercive measures such as seclusion and restraint differs very much between hospitals within a country. In 2015, a central register for all coercive measures in the German federal state of Baden-Wuerttemberg has been established for 32 hospitals treating involuntary patients. The objective of the present study was to identify factors that determine the differences between these hospitals.

**Methods:**

Data on coercive measures and diagnoses from the central register in 2015–2017 were linked with structural data of the 32 hospitals and their supply areas.

**Results:**

On average, coercive measures were applied in 6.7% of cases (*SD* = 2.8%; Min–Max = 0.35–12.0%). The proportion of affected cases was significantly correlated with the proportion of involuntary patients (*r* = 0.56), the proportion of cases with affective or neurotic, stress-related and somatoform disorders (*r* = −0.42), number of hospital beds (*r* = 0.44), a sheltered home associated with the hospital (*r* = 0.43) and number of addiction counseling centers per 100,000 inhabitants in the service area (*r* = −0.39). The final regression model only included the proportion of involuntary cases as a significant predictor (standardized beta = 0.55, adjusted *R*^2^ = 0.27).

**Conclusions:**

The predominating part of the considerable variance observed between hospitals could not be explained by structural variables. The proportion of involuntary patients had a significant impact, but a considerable amount of unexplained variance due to different practices within psychiatric hospitals remains.

## Introduction

Evidence from large-scale observational studies demonstrated that the proportion of patients affected by coercive measures varies widely between hospitals [e.g. (1–4)]. The reasons are not known so far and interpretation of these findings is an issue of considerable conflict. On the one hand, many clinicians are of the opinion that patients mainly determine the use of coercive measures (5–8). The opposite view is frequently phrased by patients' and relatives' organizations that hold attitudes and traditions among the staff responsible for differing levels of coercion (9–11). From an epidemiological point of view, such interpretation should rely on empirical findings: for example, the view that patient characteristics are important would be supported if the observed variance between hospitals could be explained mainly by patients' characteristics.

In Baden-Wuerttemberg, a German federal state with 11 million inhabitants, a central registry for all coercive measures in psychiatric hospitals and departments has been established since 2015. For cases with coercive measures, the hospitals are required to provide case-related data on the legal status of patients (i.e. staying voluntarily or being detained for treatment), primary diagnosis and duration of coercive measures. The following coercive measures have to be documented: mechanical restraint, physical restraint, seclusion and forced medication.

For the registry an online platform was set up after consultation with State Data Privacy and approval of the Data Security Officer. The platform serves both for uploading data by the institutions and for downloading data by the evaluation office.

A first evaluation showed that, in 2016, 0.3–17.5% of cases in the 32 general psychiatric hospitals treating involuntary patients were affected by coercive measures (12). We use the term “general psychiatric hospital,” clarifying thereby that forensic departments associated at some of the hospitals were not included into the analyses. As a unique feature of this registry, not only measures according to public and criminal law (for forensic psychiatry) but also according to guardianship law are recorded. The objective of this study, which is part of the research project ZIPHER: Zwangsmaßnahmen Im Psychiatrischen Hilfesystem: Erfassung und Reduktion (“Coercive Measures in the Psychiatric Help System: Recording and Reduction”) funded by the German Ministry of Health, was to link the available registry data on coercive measures with data on patients' and hospitals' characteristics and also on those of the associated supply areas. With the linked data we wanted to investigate which factors could explain differences between general psychiatric hospitals in terms of the proportion of cases subjected to coercive measures.

## Materials and Methods

### Operationalization of the Objectives

The level of coercion was measured by the proportion of patients with at least one freedom-restrictive coercive measure (seclusion, mechanical/physical restraint). This indicator can be considered reliable due to precise definitions, documentation and legal regulations for central recording (12). The following definitions are applied: Seclusion is defined as locking a person in a scarcely furnished room (mostly only with a mattress and toilet) without the presence of staff.

Mechanical restraint is defined as the use of all kinds of freedom-restricting devices, encompassing not only belts but also (undivided) bedrails, heavy blankets, nursing chairs with trays and other devices as far as they restrict free movement. Physical or manual restraint is defined as staff using physical force to hold a person.

### Case Definition in the Central Register

In the central register each complete patient treatment episode within a given reporting year is defined as a treatment case. If, for example, a patient had been admitted on 15 December 2016 and was discharged on 10 January 2017, she or he is counted in the reporting year of 2017 with all 26 days of treatment in 2016 and 2017. If a patient had been admitted on 20 December 2017 and was discharged on 5 January 2018, she or he is not counted in 2017. If a treatment episode would had lasted longer than a year it would had been counted the same way.

### Structural Characteristics of Hospitals and Supply Areas

Operationalization of the structural characteristics of hospitals and supply areas was carried out as a multi-stage process. First, the relevant structural characteristics were identified on the basis of findings from the literature and expert assessments, which were then submitted to the scientific advisory board of this study with a request for additions, proposals for changes and comments. Originally, 31 variables representing hospital characteristics and 19 variables representing characteristics of supply areas were identified. After discussion of the feedback from the scientific advisory board, two final sets of variables were prepared (46 items on hospital characteristics; 30 items on characteristics of the supply area), which were then transformed into corresponding questions (see Supplementary Material).

### Data Recording and Data Structure in the Central Register

The central register contains data on coercive measures from both forensic psychiatry and general psychiatry. Hospitals required to report data have to provide three datasets. Dataset 1 contains all the coercive measures to be reported, together with the hospital identification code, pseudonymized case number, postal code of residence, gender, main diagnosis, legal basis for hospital stay at the beginning of the coercive measure and type and duration of coercive measure. The pseudonymization of the case numbers is carried out by two separate institutions. In a first step, the hospitals encrypt the case number. Then, when uploading the data, a second pseudonymization is carried out automatically. The pseudonymization of the case numbers is thus irreversible. The other two datasets contain aggregated data on the number of treated cases, days of treatment, and legal basis for hospital stay for all cases. Dataset 1 thus contains only information on cases with coercive measures, while the datasets 2 and 3 contain information on all cases. The data are structured in such a way that the identification of specific persons is extremely unlikely: the data are de facto anonymized. This especially applies to dataset 1.

Structural characteristics of the hospitals were collected by means of questionnaires. In addition, documentation from the Local Authority Association for Youth and Social Affairs (Kommunalverband für Jugend und Soziales, KVJS) and the official quality reports of the clinics were evaluated. In order to compensate for annual random fluctuations in accommodation and coercive measures, the data for the reporting years 2015–2017 were aggregated. The structural data of the city and rural districts were assigned to the hospitals. If clinics had service areas that extended over several districts, the structural data were averaged.

### Implementation

After initial versions of the questionnaires for clinics and districts had been prepared; they were submitted to the scientific advisory board. The questionnaires were modified on the basis of feedback from the advisory board. Two questionnaires were sent out. One questionnaire, sent to the hospitals, contained 46 hospital items, the other, sent to city and county authorities contained 46 items of the supply area. The data was collected between June and September 2018. The reference times for the information were the years 2017 and 2018. Of the 32 clinics contacted, 7 refused to participate, resulting in completed questionnaires being received from 25 clinics. This corresponds to a response rate of 78%. Out of 44 city and county councils contacted, 16 counties refused to participate and thus completed questionnaires were received from 28 districts. This corresponds to a response rate of 63.6%. Additionally, the mandatory quality reports of the hospitals and the documentation from the Local Authority Association for Youth and Social Affairs were used as a basis for data evaluation. Both documentations were used to fill in missing data. In case of contradictory data, the local authorities' documentations were considered more reliable. There was no imputation of missing data. A dataset was created from data of the central register and structural data of the hospitals and supply areas. This dataset contains information on coercive measures in the years 2015–2017.

### Statistical Analyses

The data were evaluated using correlation analyses and multivariate regression models.

Data analysis was carried out using the statistics and analysis software IBM SPSS Statistics Version 27.0. To assess the correlations between variables, correlation coefficients were calculated. To assess possible predictors of the proportion of cases with coercive measures, linear regression models were fitted. Possible predictors that showed significant associations with the proportion of cases with coercive measures in bivariate analyses were entered into the models. To assess the degree to which predictors explain the variation of perceived coercion, we calculated the adjusted *R*^2^. The adjusted *R*^2^ can be interpreted as percent of variance explained. Linearity was assessed by visual inspection of the plots of observed vs. predicted values. Homoscedasticity was assessed by visual inspection of the probability–probability plot of observed vs. predicted cumulated probability of the residuals. The normal distribution of residuals was tested with a Kolmogorov-Smirnov test. Multicollinearity was tested with bivariate correlations between possible predictors.

## Results

The evaluation included data from 31 of the 32 hospitals required to provide data to the central register and from 44 city and district authorities. Data from one hospital were excluded because valid data could not be provided for all three reporting years.

**Figure 1:**
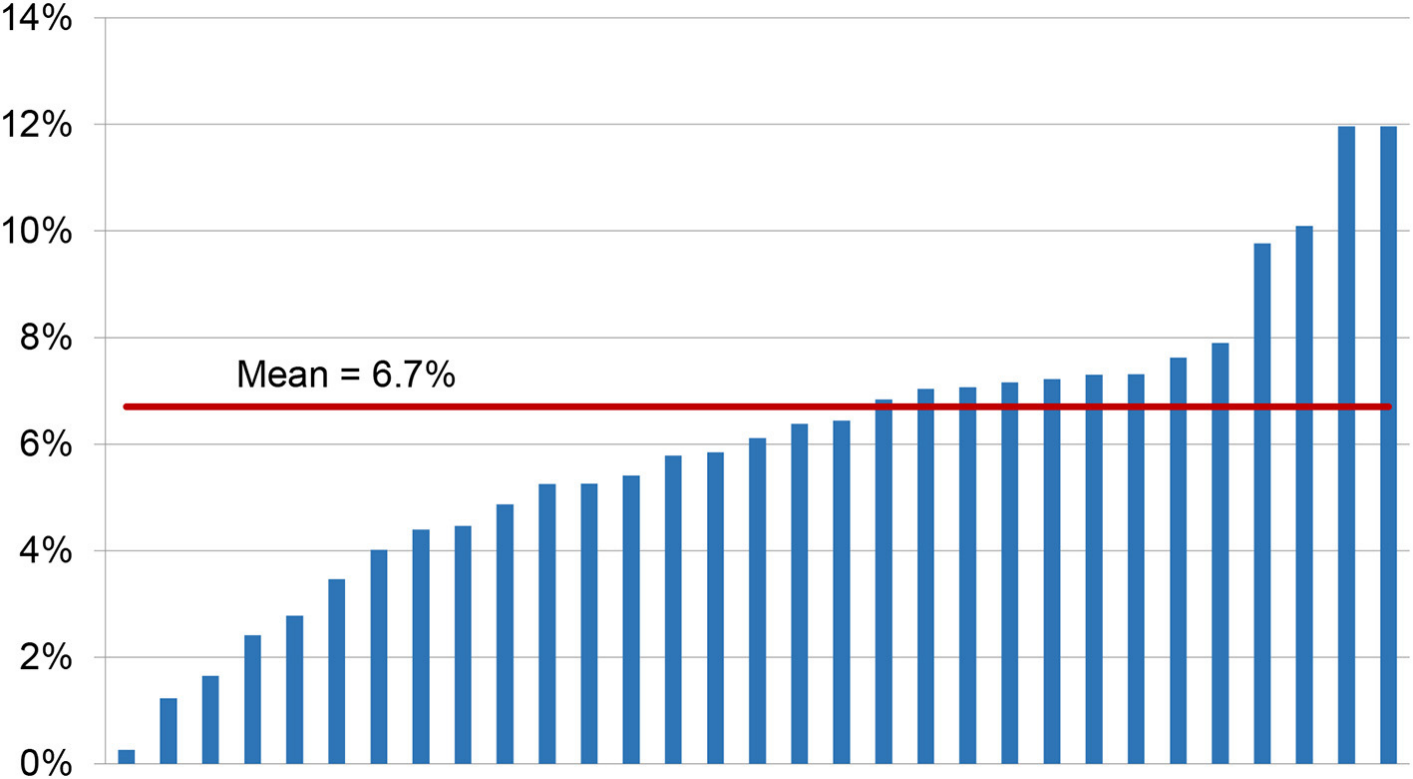
Percentage of cases with mechanical restraint or seclusion measures for the 31 hospitals in 2015–2017.

In 2015–2017 there were a total of 317,751 treated cases with 8,056,045 hospital days. There were 34,440 (10.7%) compulsory hospitalizations according to public or civil law 21,405 of cases were secluded or restrained. On average, seclusion or mechanical restraint measures were applied in 6.7% of cases (*SD* = 2.8%; Min–Max = 0.35–12.0%) (Figure 1). A total of 84,997 freedom-restrictive coercive measures were reported. The median cumulative duration of all mechanical restraint or seclusion measures per affected case was 12.7 h [Inter Quartile Range (IQR) = 28.1]. The cumulated duration of seclusion and mechanical restraint accounted for 0.5% of the length of hospital stay (Table 1).

**Table 1:** Cases with coercive measures.

	**2015–2017**
Cases	317,751
Mechanical restraints (N)	52.661
Seclusions (N)	31.881
Physical restraints (N)	494
Cases with mechanical restraints (N)	15.509
Proportion of cases with mechanical restraints	4.8%
Cases with seclusions (N)	9.157
Proportion of cases with seclusions	2.9%
Cases with physical restraint (N)	213
Proportion of cases with physical restraint	0.1%
Cases with forced medications (N)	1,902
Proportion of cases with forced medications	0.6%
Cumulated duration of mechanical restraints per affected case (Median, IQR^a^)	11.5 h (24.6)
Cumulated duration of seclusions per affected case (Median, IQR)	11.3 h (21.7)
Cumulated duration of physical restraints per affected case (Median, IQR)	0.3 h (1.2)

a *Inter Quartile Range*.

The proportion of treatment cases with freedom-restrictive coercive measures correlated significantly negatively with the proportion of patients with affective or neurotic, stress-related and somatoform disorders and with the number of addiction counseling centers per 100,000 inhabitants in the service area but correlated significantly positively with the proportion of involuntary patients and with the number of hospital beds. Clinics that operated a residential home also had significantly more coercive measures (Table 2). No other statistically significant correlations between outcome and hospital characteristics or characteristics of the supply areas were found. Table 3 shows the intercorrelations of the possible predictors.

**Table 2:** Correlation of coercive measures with possible predictors.

	**Proportion of treatment cases with freedom-restrictive coercive measures**
Proportion of patients with affective or neurotic, stress-related and somatoform disorders	−0.42*
Proportion of involuntary patients	0.56**
Number of hospital beds	0.44*
Operation of a residential home	0.43*
Number of addiction counseling centers per 100,000 inhabitants in the service area	−0.39*

* *p < 0.05*;

** *p < 0.01*.

**Table 3:** Intercorrelations of possible predictors.

	**Proportion of patients with affective or neurotic, stress-related and somatoform disorders**	**Proportion of patients with caring restraint or accommodation**	**Number of hospital beds**	**Operation of a residential home**	**Number of addiction counseling centers per 100,000 inhabitants in the service area**
Proportion of patients with affective or neurotic, stress-related and somatoform disorders	1.00	−0.41*	−0.47*	−0.19	0.47*
Proportion of patients with caring restraint or accommodation		1.00	0.37*	0.27	−0.15
Number of hospital beds			1.00	0.25	−0.09
Operation of a residential home				1.00	0.03
Number of addiction counseling centers per 100,000 inhabitants in the service area					1.00

* *p < 0.05*.

The potential predictors were included in a regression model with stepwise variable selection.

The final regression model contained only the proportion of treatment cases with involuntary admission or treatment as a significant predictor (standardized beta = 0.55, adjusted *R*^2^ = 0.27; Table 4). A Kolmogorov-Smirnov test showed no violation of normal distribution of the residuals (*p* = 0.20). The visual inspection of the plots of observed vs. predicted values showed no substantial violation of linearity, and the visual inspection of the probability–probability plot of observed vs. predicted cumulated probability of the residuals showed no substantial heteroscedasticity. The intercorrelations of the possible predictors were moderate (Table 3).

**Table 4:** Regression model.

**Final model**		**Coefficient**	**SE**	**Standardized beta**	***t***	**Significance**
	Constant	0.037	0.008		4.499	0.000
	Proportion of patients with caring restraint or accommodation	0.257	0.074	0.547	3.454	0.002
		*R* = 0.547	*R^2^* = 0.299	Adjusted *R^2^* = 0.274		
**Excluded variables**
	Proportion of patients with affective or neurotic, stress-related and somatoform disorders			−0.209	−1.226	0.231
	Number of hospital beds			0.278	1.679	0.105
	Operation of a residential home			0.298	1.897	0.069
	Number of addiction counseling centers per 100,000 inhabitants in the service area			−0.234	−1.505	0.144

## Discussion

In order to investigate contextual factors that may contribute to the use of coercive measures in inpatient psychiatric care, data from the central register of involuntary hospitalizations and coercive measures in the German federal state Baden-Wuerttemberg were linked with structural data from hospitals and supply areas. The data on involuntary hospitalizations and coercive measures in specialist psychiatric hospitals or in specialist psychiatric departments of general hospitals are unique in Germany. Since 2015, all facilities in Baden-Wuerttemberg with the authorization to involuntarily hospitalize and treat patients have been required to provide data on involuntary hospitalization and treatment to a central registration office. The data provided information on the epidemiology of coercive measures (12) and showed that there is considerable variation between hospitals in terms of the proportion of patients affected. In the present study, 0.3–12% of patients treated per hospital were affected by coercive measures. This variation cannot be further clarified with data from the central register alone. Therefore, extensive structural data of the hospitals as well as the cities and counties were combined with the data of the central register. The response rates of 78% for the hospitals and 64% for the counties can be rated as good in view of the scope and depth of the questionnaires. The bivariate correlation between coercive measures and structural characteristics showed that the proportion of treatment cases with freedom-restrictive coercive measures correlated negatively with the proportion of patients with affective or neurotic, stress-related and somatoform disorders and with the number of addiction counseling centers. These correlations appear plausible: if one assumes that in the case of affective or neurotic, stress-related and somatoform disorders there are comparatively fewer situations with acute self- or third-party danger that require the use of immediate coercion in the form of seclusion or restraint, then a negative correlation between the proportion of patients with these disorders and the use of coercion can be expected. For patients with addictive disorders who are admitted to a psychiatric ward heavily intoxicated, initial coercive measures may be unavoidable to protect the patients themselves and others. If there is a well-developed outpatient and community psychiatric support system for people with substance disorders in a service area, these patients may get help earlier and emergency admissions of severely intoxicated patients may be reduced.

A positive correlation was found for the proportion of patients who were hospitalized against their will, for the number of beds and for the operation of a residential home by a hospital. Persons who are hospitalized in psychiatric hospitals against their will represent a group of seriously ill patients with high potential for conflict due the containment they experience within the context of their involuntary stay. Against that background, the legal status of a patient and coercive measures are not independent of each other. Patients who refuse treatment and react violently when urged to take medication may experience coercive measures and subsequently a change in their legal status. The correlation between the size of a hospital (measured by the number of beds) and the proportion of patients affected by coercive measures could be explained by the fact that severely ill patients may be admitted to special wards (e.g. wards specialized for behavioral problems) that are only available in larger facilities. Another explanation could be that in large units, where a lot of personnel well-trained in coercive measures are available, many measures are also carried out, while smaller units have to rely on other (more defensive) strategies. If a clinic operates a residential home for severely chronically ill psychiatric patients, those who cannot be adequately treated in the home may be transferred to psychiatric wards in the case of acute danger to themselves or others so that coercive measures can be applied, whereas care homes without associated hospitals have to resort to other strategies to deal with emergency situations.

As structural characteristics are related to the degree of coercion in mental health facilities, it is reasonable to investigate whether these characteristics can help to explain the considerable variation between facilities. For this purpose, those structural variables that correlate with the proportion of patients with coercive measures were integrated into a regression model. Such models make it possible to estimate the simultaneous influence of several factors on a target variable. On the one hand, the chosen model should explain as much of the variation of the dependent variable as possible, but on the other hand, it should not contain any factors with negligible influence. Therefore, a step-by-step selection of variables was indicated during the model development. The final model contained only the proportion of patients hospitalized against their will as a significant predictor. With this model, 27% of the variation between institutions could be explained. The total variation between institutions can be hypothetically divided into components based on patient characteristics, hospital characteristics, characteristics of the supply area and unexplained residual variation. In the present study, regression analyses only revealed one patient characteristic contributing to the explanation of variance between hospitals in the proportion of cases with coercive measures. Yet being detained for treatment might be conceived not only as a patient characteristic but also to some degree as a characteristic of the institution. It is not completely implausible that the tradition and common practice of a hospital may affect whether, under challenging conditions, a patient is less or more likely to be detained.

Apart from the proportion of involuntary patients, no structural characteristics or characteristics of the institutions contributed to the explained variance. The extent to which almost three-quarters (60%) of unexplained variation is due to quality differences or different “treatment cultures” between the individual hospitals cannot be answered here. Physicians and nurse directors of the participating hospitals know each other well from numerous conferences and many have visited other facilities over years. However, it is still difficult for all experts to capture relevant differences in everyday procedures, which may still vary within a single hospital and over time.

The study is subject to several limitations. First of all, the small number of cases must be mentioned. The central registry provides data on more than 320,000 treatment cases, of which more than 34,000 are involuntary cases and more than 21,000 are treatment cases with coercive measures. However, because the level of analysis is the individual hospital, the number of cases for statistical modeling is reduced to 31. The number of cases and the statistical significance of influencing variables are now coupled in such a way that, as the number of cases increases, significance is achieved even for small effects. Thus, it cannot be excluded that different results would be found if other hospitals were included. On the other hand, even larger comparable studies from abroad do not clearly show a substantial influence of structural features (1, 7).

Another limitation is that the proportion of cases being detained for treatment is affected by the use of coercive measures. In some cases, patients who are initially voluntarily admitted may experience a coercive measure in the course of treatment, which in turn mandatorily changes the legal status to “involuntary.”

A further limitation of the study is that ward characteristics were not recorded. This would have been possible at great expense for the survey with the questionnaires but not for the central register. The register contains no information on any characteristics of wards.

For data protection reasons, the central register contains only minimal personal data that have been anonymized. For ward characteristics as well as for patient characteristics, however, it can be assumed that further information could increase the proportion of explained variation (5, 6, 13).

Finally, an exact allocation of the service areas of the hospitals and the city and county districts was not always possible. Regional structural data were only available at the district level, whereas service areas can extend beyond district borders. Hospitals do not always provide their services to entire districts, therefore the structural data of several districts were averaged, if necessary.

## Conclusion

The predominating part of the considerable variance observed between hospitals could not be explained by structural variables. The proportion of involuntary patients had a significant impact, but a considerable amount of unexplained variance due to different practices within psychiatric hospitals remains.

## Data Availability Statement

The data analyzed in this study is subject to the following licenses/restrictions: The datasets generated for this study cannot be made publicly available. The data stored in the register is classified as confidential by the data protection officer of Baden-Wuerttemberg and is not publicly available due to data privacy. Requests to access these datasets should be directed to erich.flammer@zfp-zentrum.de.

## Author Contributions

TS made substantial contributions to the conception, design of the work, introduction, the interpretation of data, and the discussion. NT contributed to the conception, design of the work, and collected data. SH substantially contributed to the introduction, the interpretation of data, the discussion and critically revised the manuscript. EF substantially contributed to the conception, design of the work, contributed to the introduction, the interpretation of data, the discussion, was responsible for the methods, made the statistical calculations, substantially contributed to the results, and contributed to the discussion. All authors contributed to the article and approved the submitted version.

## Funding

This study was part of the research project ZIPHER: Zwangsmaßnahmen Im Psychiatrischen Hilfesystem: Erfassung und Reduktion (Coercive Measures in the Psychiatric Help System: Recording and Reduction) funded by the German Ministry of Health (ZMVI-2515FSB406).

## Conflict of Interest

The authors declare that the research was conducted in the absence of any commercial or financial relationships that could be construed as a potential conflict of interest.

## Publisher's Note

All claims expressed in this article are solely those of the authors and do not necessarily represent those of their affiliated organizations, or those of the publisher, the editors and the reviewers. Any product that may be evaluated in this article, or claim that may be made by its manufacturer, is not guaranteed or endorsed by the publisher.
