# Supplementary material for: “Our Patients Are Different”: Predictors of Seclusion and Restraint in 31 Psychiatric Hospitals
Source: Front Psychiatry. 2022 Apr 26;13:791333. doi: 10.3389/fpsyt.2022.791333 (PMC9086971; doi:10.3389/fpsyt.2022.791333)
Supplement: Supplementary file 1 [file Table_1.DOCX]

**Supplement: Questionnaires assessing the characteristics of the hospitals and of the service areas**

**Questionnaire assessing the characteristics of the hospitals**

| **General questions about the characteristics of the hospital** | Is the hospital a university hospital, a specialist hospital or is it a specialist department/clinic at a general hospital/ children's hospital? |
| --- | --- |
|  | Does the clinic have a regional service obligation for psychiatry? |
|  | Is the hospital in public ownership? |
|  | Which specialist psychiatric departments are available? |
| **Questions about quantitative and qualitative key figures of the hospital** | How many beds/places per 100,000 inhabitants of the district are available for inpatients and day patients (listed separately for specialist departments)? |
|  | How many cases are treated (inpatients and day patients; listed separately for specialist departments)? |
|  | Is there complaint management in the hospital? |
| **Questions about characteristics and qualifications of the hospital staff** | What is the number of full-time positions of (specialist) doctors, nursing staff, psychologists and specialist therapists per bed/place listed separately by department? |
|  | What proportion of doctors and psychologists received specialist training? |
|  | What proportion of doctors and psychologists are licensed as psychotherapists? |
|  | What proportion of doctors and psychologists are licensed as child and youth psychotherapists? |
|  | What is the ratio of fully qualified nurses with three years' training to auxiliary staff, excluding trainees and students? |
|  | For child and adolescent psychiatry: What is the ratio of fully trained staff to auxiliary staff – without interns (fully trained child health and nursing staff, educators and social workers in ward service)? |
| **General questions about the characteristics of the hospital** | From which catchment area (urban districts and/or counties) are patients admitted or treated? |
|  | Does the clinic run nursing homes? |
|  | Is the clinic management represented in committees of community mental health services? |
| **Question about patient-specific characteristics of the hospital** | What is the percentage of cases with F3/F4 main diagnoses? |
| **Questions about support services of the hospital** | Are peer workers employed on the wards (also acute wards)? |
|  | Can the clinic organize a qualified interpreting service within 24 hours in the following languages: Arabic, English, Farsi, French, Italian, Persian, Polish, Russian, Serbo-Croatian, Spanish, Ukrainian |
|  | Does the clinic offer home treatment? |
| **Questions about the situation at admission** | Are there any rooms other than one- or two-bed rooms at the hospital‘s admission wards? |
|  | For child and adolescent psychiatry: Is there a spatially separate crisis/admission area for children and adolescents? |
|  | For child and youth psychiatry: Is there an emergency and standby service of the youth welfare office? |
|  | For child and adolescent psychiatry: Is it possible to have adolescents taken into care by the youth welfare office at the end of treatment or instead of admission? |
|  | Is there an interdisciplinary emergency room? |
|  | Is there a central crisis or intensive care unit? |
|  | What is the proportion of patients who are transferred to another ward during their hospital stay? |
|  | Do the admission wards offer the opportunity to accommodate patients who could be a burden for fellow patients in a single room? |
|  | For child and adolescent psychiatry: Is there disclosure of possible coercive measures to patients and guardians at admission? |
| **Question about the structural equipment/architecture of the hospital** | Is seclusion carried out at the hospital without simultaneous restraint? |
| **Questions about specific characteristics of the hospital with respect to coercive measures** | Are there compulsory de-escalation training courses for staff? |
|  | Is an internal guideline for coercive measures accessible to all employees? |
|  | Does the clinic have a specialized or mixed concept? |
|  | Has there been a new clinic construction/conversion in the acute area in the last 10 years? |
|  | Are there (except forensic psychiatry) continuously closed wards? |
|  | Does the clinic have supra-regional special wards with the application of coercive measures (e.g. for intellectually disabled people)? |
|  | Is there a specific strategy with specific programs to reduce coercive measures? |
|  | What is the proportion of involuntary admission and treatment in the total admissions? |
|  | How long do emergency patients need on average to get to the hospital? |
|  | How often is a joint-crisis plan actually used in the hospital per year? |
|  | Is there a patient advocate who can also be reached if a patient is treated on a closed ward? |
|  | Where are geriatric patients with delirium taken care of? |
|  | Where are patients with addiction disorders and delirium taken care of? |
|  | Where are aggressive and drunk patients typically taken care of? |
| **Questions about the judicial hearing in the hospital** | In your experience, how long does it take on average until a judicial hearing takes place? |
|  | Is there an on-call service at weekends for a judicial hearing? |

**Questionnaire assessing the characteristics of the service areas**

| **General questions on the characterization of the county/urban district** | How large is the area of the county/city district and how many inhabitants does it have? |
| --- | --- |
|  | What is the population structure of the county/city district in terms of age, gender and proportion of minorities and immigrant groups? |
|  | What is is the unemployment rate in the county/city district? |
|  | For child and youth psychiatry: What is the youth unemployment rate in the county/city district? |
|  | What is the poverty rate in the county/city district (at-risk-of-poverty rates based on the state median)? |
|  | For child and youth psychiatry: What is the child poverty rate in the county/city district (at-risk-of-poverty rates of under 18-year-olds on the basis of the state median)? |
|  | What is the ratio of persons receiving social assistance to the working age population in the county/city district? |
|  | What is the proportion of persons receiving social assistance for jobseekers with children in the county/city district? |
|  | What is the crime rate in the county/city district (number of crimes in the county/city district)? |
|  | For child and youth psychiatry: What is the youth crime rate in the county/city district (number of suspects under 21 years of age/total offenses in the county/city district)? |
| **Questions about the mental health services offered by the county/city** | How many self-help groups and contact clubs for mentally ill people are in the county/city district? |
|  | How many counseling centers for mentally ill persons are in the county/city district? |
|  | Are there day-care centers for mentally ill people in the county/city district? |
| **Questions about housing offers for mentally ill people in the county/city district** | How many places for outpatient assisted living are in the county/city district (alternatively for other federal states, the number of persons receiving outpatient integration assistance for independent living)? |
| **Questions about the psychiatric services offered by the county/city** | Does helping to plan conferences take place in the county/city district? |
|  | Are there one or more community mental health services? |
|  | For child and youth psychiatry: Do conferences with possible patient/family participation take place in the county/city district, involving the youth welfare office, school board, employment agency and social assistance providers? |
|  | For child and adolescent psychiatry: How many self-help groups for adolescent patients and parents are in the county/city district? |
|  | For child and youth psychiatry: How many youth welfare institutions are in the county/city district? |
|  | Is there an outpatient psychiatric nursing service in the county/city district? |
|  | How many services that provide ‘Soziotherapie’ (service helping mentally ill people to receive medical and complementary therapies) are in the county/city district? |
|  | How many addiction counseling centers are in the county/city district? |
|  | How many low-threshold contact and counselling services for people with substance use problems exist in the country/city (contact points, legal/illegal drugs, clean/on drugs)? |
| **Questions about housing offers for mentally ill people in the county/city district** | Are there residences for mentally ill people? |
|  | How many people can be cared for in closed residential homes in the county/city district? |
|  | For child and youth psychiatry: How many closed youth care places are there in the county/city district? |
|  | How many residents of residential homes originally come from the county/city district in which the residential home is located and how many come from other county/city districts? |
| **Questions on forensic aspects/ patients in the county/city district** | Do former forensic patients live in residential homes in the county/city district? |
|  | For child and adolescent psychiatry: Is there a forensic department of child and adolescent psychiatry in the county/city district? |
|  | Is there a forensic psychiatric hospital in the county/city district whose discharged patients regularly stay and receive care in the county/city district? |
